# G-Quadruplexes in Pathogens: A Common Route to Virulence Control?

**DOI:** 10.1371/journal.ppat.1004562

**Published:** 2015-02-05

**Authors:** Lynne M. Harris, Catherine J. Merrick

**Affiliations:** Centre for Applied Entomology and Parasitology, School of Life Sciences, Keele University, Keele, Staffordshire, United Kingdom

## Abstract

DNA can form several secondary structures besides the classic double helix: one that has received much attention in recent years is the G-quadruplex (G4). This is a stable four-stranded structure formed by the stacking of quartets of guanine bases. Recent work has convincingly shown that G4s can form in vivo as well as in vitro and can affect both replication and transcription of DNA. They also play important roles at G-rich telomeres. Now, a spate of exciting reports has begun to reveal roles for G4 structures in virulence processes in several important microbial pathogens of humans. Interestingly, these come from a range of kingdoms—bacteria and protozoa as well as viruses—and all facilitate immune evasion in different ways. In particular, roles for G4s have been posited in the antigenic variation systems of bacteria and protozoa, as well as in the silencing of at least two major human viruses, human immunodeficiency virus (HIV) and Epstein-Barr virus (EBV). Although antigenic variation and the silencing of latent viruses are quite distinct from one another, both are routes to immune evasion and the maintenance of chronic infections. Thus, highly disparate pathogens can use G4 motifs to control DNA/RNA dynamics in ways that are relevant to common virulence phenotypes. This review explores the evidence for G4 biology in such processes across a range of important human pathogens.

## What Are G-Quadruplexes and Why Are They Important?

Over a hundred years ago it was reported that concentrated guanylic acid can self-assemble [1], but it was not until the 1960s that the structural basis for this phenomenon, the G4, was elucidated [2]. G4s were initially considered a structural curiosity; however, it has since become clear that they are involved in a number of key biological functions. This has led to the emergence of G4s as a hot topic in nucleic acids research with the vast majority of this research thus far undertaken in highly tractable model systems such as *Saccharomyces cerevisiae* or human cell lines. However, there is now a rapidly developing literature on the roles of G4s in human pathogens. This review will briefly outline the known roles for G4s in the cell biology of model systems, and then explore how these can map onto pathogen biology, particularly to facilitate immune evasion.

**Fig 1:**
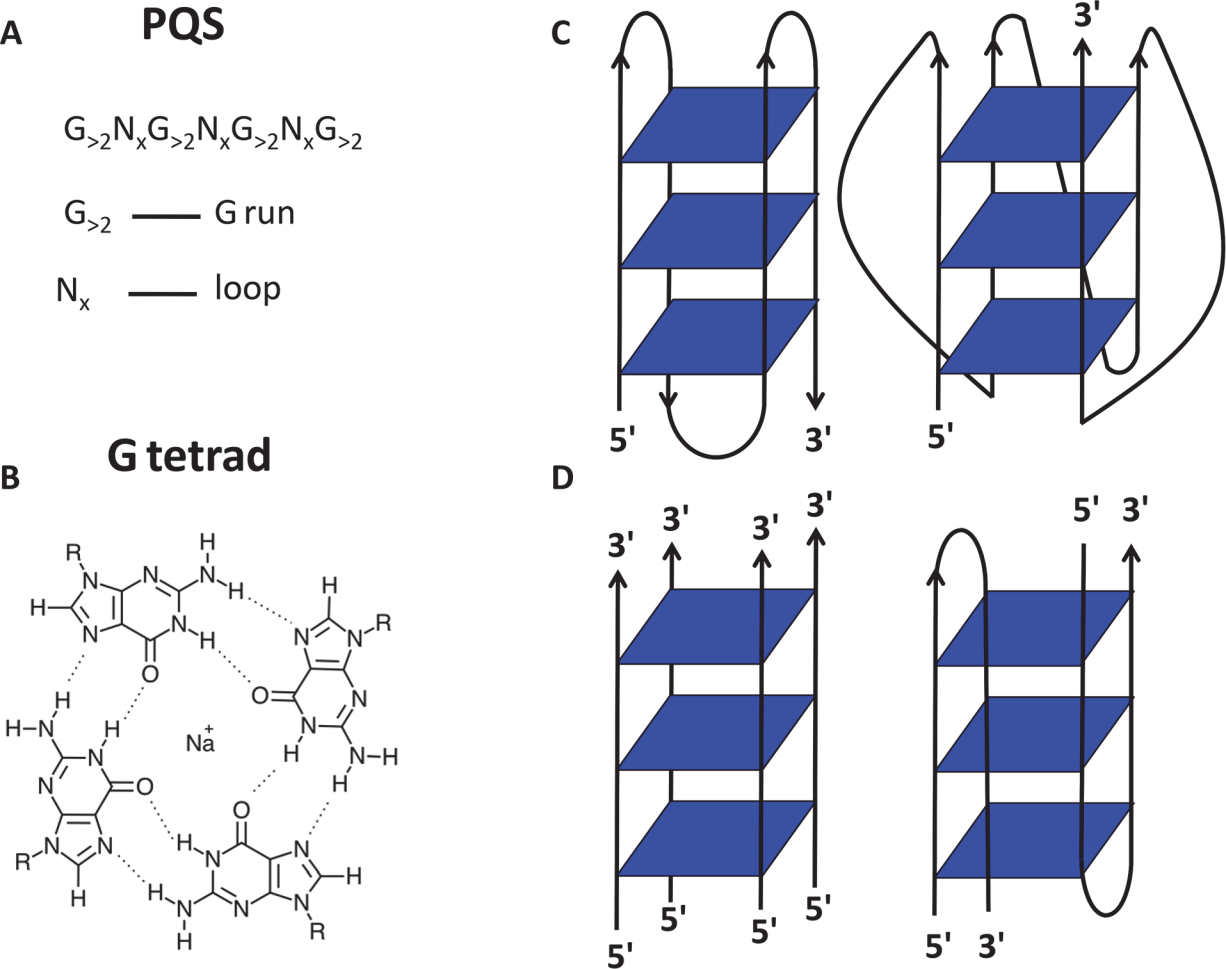
G-quadruplex (G4) structure. (A) A putative quadruplex sequence (PQS) is a nucleotide sequence predicted to form a G4 structure. A degenerate PQS used to predict the formation of intramolecular G4s is shown here, consisting of four runs of at least three guanines per run, separated by short stretches of other bases (N). (B) The basic unit of the G4 is the G-tetrad. (C) G4 structures display a large variety of different topologies. Topology of intramolecular G4 structures displaying antiparallel (left) and parallel (right) configurations. (D) Topology of intermolecular G4 structures formed by dimerisation of four strands (left) or two strands (right).

In terms of structure, the basic unit of the G4 is the G-tetrad, a planar array of four Hoogsteen-bonded guanine bases, which when stabilised by monovalent cations, stack on top of one another to form the G4 structure itself (Fig. 1). The stacked G-tetrads are connected by loops formed from intervening mixed-sequence nucleotides: these loops vary in both size and sequence from one G4 to another. The four strands comprising the G4 may originate from one, two, or four separate strands of DNA or RNA. G4s can therefore be described as either intramolecular or intermolecular (Fig. 1C, D). In addition, there is directionality to the strands, which can be described as running from the 5′ end to the 3′ end. G4s can therefore exist as a number of topological variants (Fig. 1C, D). The conformation of glycosidic bonds of guanine bases in G-tetrads, the cations present and the number of stacked G-tetrads further contribute to the myriad of topologies found amongst G4s [3–6].

Predictive algorithms, such as G4P Calculator [7] and QuadParser [8], have been developed to identify putative quadruplex sequences (PQS) within nucleic acid sequences. Use of these algorithms in whole genome sequences has revealed that PQS are not randomly located throughout genomes but are overrepresented in gene regulatory regions and repetitive regions such as telomeres [9,10]. RNA G4s are present in transcripts associated with telomeres, in noncoding regions of primary transcripts, and also in mature transcripts.

The regions in which PQS occur are linked to the specific functions of G4s at these locations. For example, at telomeres, G4 structures are proposed to be involved in telomere maintenance at both the RNA and DNA level. Eukaryotic telomeric DNA consists of long stretches of tandemly repeated G-rich sequences, such as GGGTTA in humans, which end in a 3′ single-stranded DNA overhang. A protein complex caps these overhangs in order to prevent them being identified by cellular surveillance mechanisms as unwanted DNA breaks. These G-rich telomeric repeats can form G4s both in vitro and in vivo and can protect telomeres: in *S*. *cerevisiae* telomeric G4s may provide an alternative form of telomere capping when natural capping is compromised [11]. In addition, telomeric G4s protect the telomeric 3′-overhang from being recognised by telomerase, thereby regulating telomerase activity. Human telomeres are transcribed to produce long, noncoding telomeric repeat-containing RNAs (TERRAs), which consist of UUAGGG repeats and adopt a G4 RNA structure [12,13]. TERRAs interact with the telomere binding protein TRF2 to promote telomere heterochromatinisation [14,15].

In promoter regions, the dynamic behaviour of G4s may be directly involved in gene regulation at the level of transcription. One of the best studied systems for a role of G4s in transcriptional regulation is the human *c-MYC* locus. c-MYC is a transcription factor whose expression is linked to cell proliferation and tumourigenesis. Nuclease hypersensitive element III (NHE III_1_), a major regulator of *c-MYC* transcription, contains a PQS that forms a G4 structure in vitro [16]. A gene containing wild-type NHE III_1_ is less expressed than one containing a mutated version that cannot form a G4 structure, so the PQS in NHE III_1_ represses transcription [17]. Additionally TMPyP4, a G4-stabilising ligand, reduces c-MYC expression in lymphoma cell lines [17].

Where G4s occur in gene bodies, they can form steric road blocks to the DNA transcriptional machinery, and certain helicases, such as those of the RecQ and Pif1 families, possess G4-resolving activity to aid transcription through these four-stranded snags. Disruption of G4-resolving helicases in *Caenorhabditis elegans* [18], *S*. *cerevisiae* [19] or human cells lines [20] results in genetic instability, and G4s are a hallmark of fragile sites in the human genome [21]. In RNA transcripts, G4s may play roles in pre-mRNA processing, translation and RNA turnover.

Given their propensity to induce genomic instability and DNA damage, it is hypothesised that G4s could be used alongside inhibitors of DNA repair or associated pathways to inhibit tumourigenesis. For example, the G4-interactive compound Quarfloxin (CX-3543, Cylene Pharmaceuticals) is a first-in-class drug that progressed to Phase II clinical trials for cancer. Quarfloxin selectively disrupts the interaction of ribosomal DNA G4s with nucleolin, thereby inhibiting RNA polymerase I and inducing apoptosis in cancer cells [22]. Furthermore, stabilisation of G4s may be an effective approach by which to inhibit telomerase activity in tumour cells [23]. The field of G4 biology, so far largely motivated by this potential for developing novel anti-cancer therapeutics, has provided us with both a conceptual basis and a molecular toolset (Box 1) with which to approach the burgeoning field of pathogen G4 biology.

**Box 1. The G4 Biology Toolbox**

Many tools have been developed to analyse G4s at both genome-wide and sequence-specific levels.

**Predictive algorithms**

Intramolecular G4s form from short runs of guanine bases separated by short runs of other bases. Intermolecular G4 structures form from G-runs on two or more nucleic acid strands. Therefore, by searching for sequences matching these criteria, we can identify genomic regions that may form G4s, termed putative quadruplex sequences (PQS) or G4 motifs. Predictive algorithms (reviewed in [70]), including quadparser [8], G4P calculator [7], QGRS mapper [71] and QuadBase [72], have been developed to identify PQS. These algorithms can rapidly analyse large amounts of data, including whole genome sequences.

**Biophysical techniques**

Several experimental techniques can determine whether the biophysical features of a synthetic PQS-containing oligonucleotide are consistent with G4 structure formation. These include dimethylsulfate footprinting, thermal denaturation, ultraviolet spectroscopy and circular dichroism spectroscopy. Complete structure determination necessitates the use of X-ray crystallography or nuclear magnetic resonance structure determination.

**G4-interactive compounds**

Diverse families of small molecules preferentially bind to G4 DNA over other types of DNA. These include the fluoroquinolone quarfloxin [22], the acridine derivative BRACO19 [73], telomestatin [74], and triazines [75], among many others (reviewed in [4]). Compounds that stabilise and/or induce G4 formation can be used to investigate the biological roles of G4s. In addition, the RNA G4-selective ligand carboxyPDS has recently been described [69].

**G4 structure-specific antibodies**

G4-structure specific antibodies have been exploited to visualise G4s in both genomic DNA and cytoplasmic RNA. High-affinity single-chain antibodies, generated by ribosome display, have been used to visualise G4s at the telomeres of the ciliate *Stylonychia lemnae* [76,77]. More recently, a monoclonal single-chain antibody generated by phage display has been used to visualise G4s in the genomic DNA and cytoplasmic RNA of a range of mammalian cells [68,69].

**G4-interacting proteins**

Many proteins have been identified which interact physically and/or functionally with G4s in a variety of organisms. Some G4-interacting proteins, such as the β subunit of the *Oxytricha nova* telomere-binding protein, promote the formation of G4s from PQS [78,79]. The binding of some proteins to extant G4s increases the stability of the G4 structure [80,81]. Conversely, a group of G4 destabilising proteins has been discovered. These include the highly conserved human telomeric protein POT1 [82] and a number of single-strand binding proteins of the heterogenous nuclear ribonucleoprotein (hnRNP) family [83–85]. In addition a number of helicases preferentially bind and disrupt DNA G4s. Experimentally modulating the expression or stability of these proteins offers a means by which to perturb G4 stability.

## Roles for G4s in Recombination-Mediated Antigenic Variation

Antigenic variation (Av) is the process by which pathogens express different versions of their surface epitopes in order to evade detection by the host immune system. In general, the pathogen possesses a bank of genes encoding possible antigenic variants but expresses only one of these genes at any time. Switching can be mediated by a variety of sophisticated genetic and epigenetic systems, but in several well-characterised pathogens—including *Neisseria gonorrhoeae*, the bacterium responsible for gonorrhoea, and *Trypanosoma brucei*, the causative agent of sleeping sickness—it is genetic recombination into a single gene expression site that mediates antigenic switching.

**Fig 2:**
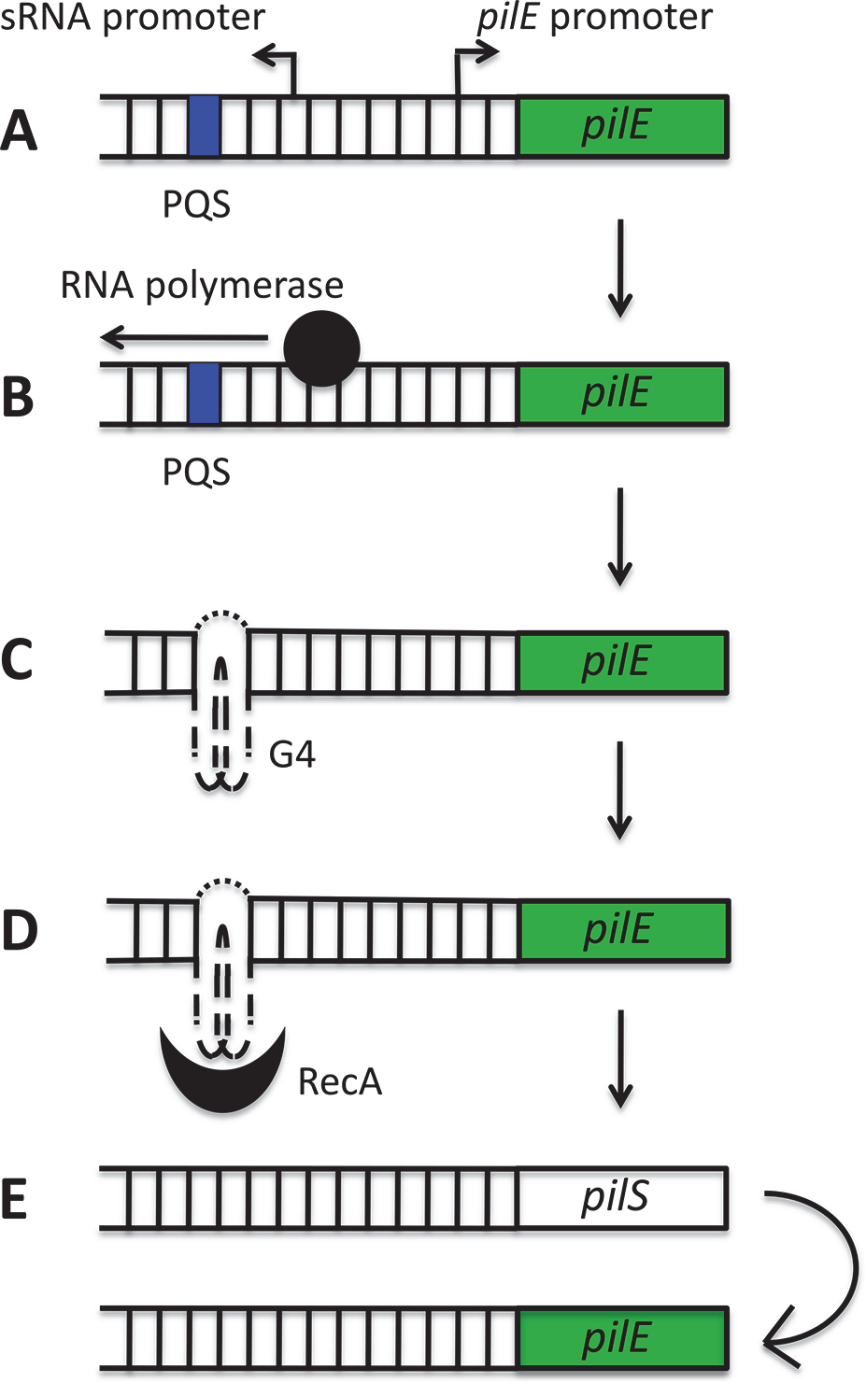
Schematic model of the role of the *pilE* G-quadruplex (G4) in *N*. *gonorrhoeae* pilin antigenic variation. A putative quadruplex sequence (PQS) and a small RNA (sRNA) promoter are located upstream of the *pilE* locus (A). The initiation of transcription from the sRNA promoter (B) provides the single-stranded conditions required for G4 formation (C). The *pilE* G4 recruits RecA (D) and potentially other recombination factors, which stimulates non-reciprocal recombination between a *pilS* locus and the *pilE* locus (E).

G4s have now been implicated in Av in several pathogens, the best characterized example being *N*. *gonorrhoeae*, which uses a G4-mediated system to switch the expression of its cell-surface pilin proteins (Fig. 2). During pilin antigenic variation, only the pilin gene residing at the active *pilE* locus is expressed and the resident gene is frequently replaced with a gene from a pool of silent *pilS* loci. The recombination initiation site has been mapped to a 16 base pair (bp) G-rich segment that forms a parallel intramolecular G4 structure in vitro and is located upstream of the *pilE* locus [24]. Disruption of this structure through site-directed mutagenesis prevented the formation of single-stranded nicks that can initiate recombination, and thus suppressed pilin Av [24]. Furthermore, recombinant RecA specifically bound to the *pilE* G4 to stimulate strand exchange in vitro, suggesting that the G4 structure may recruit recombination factors to the *pilE* locus [25]. Finally, mutation of the *N*. *gonorrhoeae* RecQ helicase (a structure-specific helicase which can unwind the *pilE* G4 structure in vitro) resulted in defective pilin Av [26].

A long-standing question in the field of G4 biology has been whether these structures actually form and persist in vivo from the inherently stable DNA double helix. For this to occur, G4 structure formation must be preceded or accompanied by localised unwinding of the double helix. Active transcription results in the spread of negative superhelicity behind the transcriptional machinery [27,28], and this can provide the torsional stress required for unwinding of the double helix and the formation of G4s [29]. At the *pilE* locus, active transcription of a small noncoding RNA (sRNA), which initiates within the *pilE* G4-forming sequence, is required for pilin Av (Fig. 2B). The expression of this sRNA in *trans* cannot complement a loss-of-function mutant in the sRNA promoter, implying that active in situ transcription may be providing the torsional stress required for helix unwinding and G4-structure formation [30].

It is interesting to speculate on whether this well-characterised system for promoting recombinational Av can be generalized to other pathogens. A recent analysis of >600 *Neisseria meningitidis* isolates found PQS adjacent to some, but not all, *pilE* gene sequences; specifically, they were found 5′ or 3′ of class I, but not class II, *pilE* loci. [31]. Given that the former, and not the latter, are antigenically unstable, we speculate that G4s may also play a role in *N*. *meningitidis* class I pilin Av.

An entirely different genus of pathogenic bacteria also evades the host immune response through Av: these are the spirochetes *Borrelia* spp., some of which cause human Lyme disease. Lyme spirochetes evade the host immune response through gene conversion-driven Av of a surface lipoprotein, Vmp-like sequence E (VlsE). Runs of guanines capable of forming intermolecular G4 structures in vitro are abundant on the coding strand of the *vls* locus in several Lyme *Borrelia* strains and species [32]. It is not yet known whether G4s actually form in vivo at this locus to play an active role in promoting gene conversion. In vitro, however, *Borrelia* telomere resolvase does appear to disrupt intermolecular G4s formed from this locus [33]. This enzyme has single-strand annealing and strand exchange activities, both of which have been hypothesised to play roles in gene conversion at the *vlsE* locus.

Further understanding of the initiating events of antigenic switching in this system will help to determine what, if any, role G4s play in Lyme *Borrelia* Av and recombinational switching. More generally, genome-wide scans for the distribution of PQS within bacterial genomes suggest that G4s are widely distributed and may actually have quite broad roles in the regulation of bacterial genes. G4s may therefore affect virulence processes in many more bacterial pathogens, and tractable bacterial species that contain G4s, like *Escherichia coli*, could prove useful models for future studies [34,35]. Finally, this model may also apply in eukaryotic as well as prokaryotic microbes, since there is an unexplored role for G4s in the antigenic switching of variant surface glycoproteins in *T*. *brucei*, where G-rich telomeres—potential G4-forming sites—facilitate the switching of VSG virulence genes [36].

## Other Roles for G4s in Antigenic Variation

It is clear that G4 motifs have key roles in certain recombination-mediated Av systems. There are, however, other systems of Av that are mechanistically distinct, such as that mediated by epigenetic silencing in the fungal pathogen *Candida glabrata* [37] and the protozoan malaria parasite *Plasmodium falciparum* [38]. Could G4s play a role here too? Although this area remains underexplored, bioinformatic work on *P*. *falciparum* suggests that indeed they could. A search of the *P*. *falciparum* genome for G4 motifs revealed that outside of the telomeres, PQS are rare in this AT-rich genome [39]. Interestingly, half of the non-telomeric PQS are located in or upstream of a large, multicopy, hypervariable gene family called *var* [39]. *Var* genes encode *P*. *falciparum* erythrocyte membrane protein 1 (PfEMP1), a family of variant immunodominant surface antigens, which are important virulence factors [40–42]. *Var* gene expression is mutually exclusive and *P*. *falciparum* frequently switches to express different *var* genes. Furthermore, *var* genes regularly recombine to generate new variants. Transcriptional switching is mediated by epigenetic silencing of all but one *var* locus and switching occurs when the current active site becomes heterochromatic while another site becomes euchromatic and actively transcribed—the trigger for this is still unknown [43].

The predominance of G4 motifs in *var* gene regulatory regions suggests that they may play roles in *var* gene recombination and/or switching. G4 motifs upstream of well-characterised genes in model systems can alter levels of transcription, e.g., in the *c-MYC* oncogene [17], and changes to G4 metabolism can also induce switches in epigenetic silencing [44–46]. *Plasmodium* possesses at least some of the enzymes known to metabolise G4s: *P*. *falciparum* contains two putative RecQ helicases [47], although there is no recognizable Pif1. It is also notable that most of the *var* family (like several other variantly-expressed gene families in this parasite) is located just inside the telomeres, raising the possibility that telomeric G4s may play roles in both telomere maintenance and the regulation of subtelomeric virulence gene families. Synthetic oligonucleotides composed of the degenerate *P*. *falciparum* telomeric motif GGGTTYA are indeed able to form stable G4 structures in vitro [48], although their biological significance awaits confirmation in vivo.

## Could G4s Play a Role in Viral Latency?

**Fig 3:**
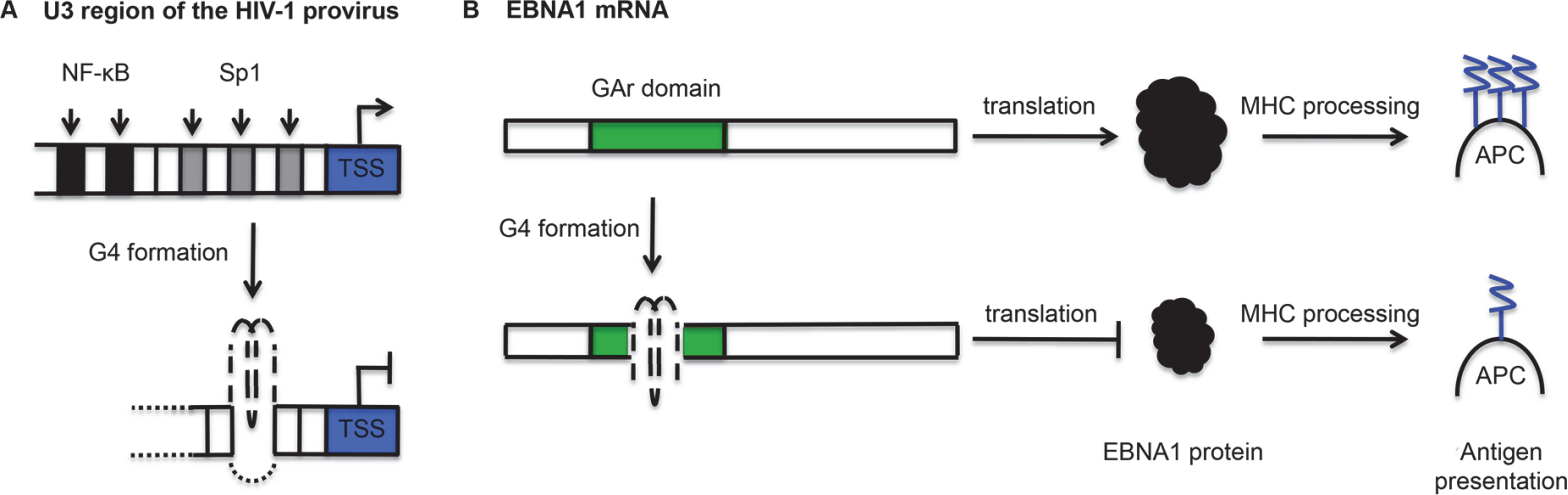
Schematic representation of the mechanisms by which G-quadruplexes (G4) may contribute to the maintenance of viral latency. (A) In the integrated HIV-1 provirus, the U3 region contains two NF-κΒ binding sites (black blocks) and three Sp1 binding sites (grey blocks). Here, the formation of a G4 from the G-rich Sp1 binding sites is shown, but several distinct G4s can form in this region. G4 formation inhibits transcription from the transcription start site (TSS). This transcriptional repression may be mediated by the differential affinity of transcription factors for double-stranded and G4 DNA. (B) The glycine-alanine repeat domain of Epstein-Barr virus-encoded nuclear antigen 1 (EBNA1) mRNA contains PQS. G4 formation inhibits translation, thereby reducing EBNA1 protein levels and limiting the presentation of EBNA1 peptides by antigen presenting cells (APC) via the major histocompatibility complex (MHC) pathway.

Recent research suggests that viruses may use G4s as *cis*-acting regulatory elements in gene expression. The retroviral HIV-1 genome contains two copies of single-stranded RNA encoding nine genes. Following infection, the RNA is transcribed by reverse transcriptase into double-stranded DNA, which is integrated into the infected cell genome. The HIV-1 long terminal repeat (LTR) controls viral transcription within the integrated provirus and contains two intramolecular G4s within a 57 bp G-rich tract of its U3 promoter region [49]. This tract contains five cellular transcription factor binding sites: two NF-κB and three Sp1 (Fig. 3A). When the wild-type LTR sequence was placed upstream of a luciferase reporter, G4-disrupting mutations or the addition of a G4-stabilising ligand resulted in increased and decreased promoter activity, respectively, suggesting that G4s may regulate HIV-1 LTR promoter activity [49].

There is further evidence that the U3 promoter region may be capable of dynamically adopting various G4 structures with the potential to bind cellular transcription factors. In addition to the two parallel-like intramolecular G4s described above, Amrane and colleagues found that a synthetic sequence derived from the same promoter region could form a hybrid G4 structure consisting of a two-G-tetrad antiparallel G4 with a further Watson-Crick CG bp [50]. Furthermore, Piekna-Przybylska et al. found that synthetic oligonucleotides comprising G-runs from the three Sp1 binding sites could adopt a variety of configurations in vitro, including parallel, antiparallel and hybrid G4 conformations [51]. Pull-down assays indicated that Sp1 could bind to one of the Sp1 sites folded into a G4 structure [51]. Given that Sp1 activity is associated with the maintenance of viral latency [52,53] and G4 structures within the U3 promoter region can suppress transcriptional activity [49], this potential for alternate protein-DNA interactions may play a role in HIV-1 latency [51].

G4s may also play a role in the transcriptional regulation of the HIV-1 provirus itself. Three adjacent, highly conserved PQS, capable of forming stable G4 structures in vitro, are located within the proviral *nef* gene. When the *nef* gene was cloned upstream of the reporter gene green fluorescent protein (GFP), the addition of a G4-stabilising drug, but not of its non-G4 binding analogue, reduced GFP expression levels [54]. This G4-mediated transcriptional regulation may have important implications for viral pathogenicity as the HIV-1 Nef protein is an important virulence factor [55]. Finally, stable G4 structures can form in regions of the HIV-1 RNA genome [51,56], where they may be involved in regulating reverse transcription of the genome.

Moving from HIV-1 to EBV, very recent evidence suggests that G4s may also play a role in the latency program of this gammaherpesvirus. Here, however, the G4 acts at the level of RNA translation rather than DNA transcription. Following infection, down-regulation of viral protein synthesis relies heavily upon a class of viral proteins that can inhibit their own synthesis, termed “genome maintenance proteins.” The mRNA for one of these genome maintenance proteins, EBV-encoded nuclear antigen 1 (EBNA1), contains G4 structures within its glycine-alanine repeat domain (GAr)-encoding region. These may regulate its translation, since disruption of these G4 structures reduces ribosome dissociation and increases EBNA1 mRNA translation (Fig. 3B) [57]. Furthermore, destabilisation of these G4 structures, following transfection of a vector expressing native GAr mRNA fused to a sequence encoding an ovalbumin epitope, resulted in enhanced antigen presentation of that epitope by a T-cell hybridoma [57]. These results have been mimicked with the EBNA1 antigen itself in an in vivo mouse model, where EBNA1 mRNA G4 destabilisation resulted in enhanced antigen presentation by dendritic cells and the early priming of CD8+ T cells [58], demonstrating that reducing the translation efficiency of viral proteins is a key part of the latency program of this gammaherpesviruses.

Finally, G-rich regions from both control and coding regions of some human papillomavirus (HPV) genomes are able to form G4 structures and may also carry out regulatory functions in this virus [59]. The roles of G4s in the silencing of at least two major human viruses provide them with a flexible tool with which to evade immune detection and thereby maintain chronic infections. Thus, although the mechanisms are entirely distinct, the results in terms of virulence are comparable to those of the Av systems described above.

## Roles for G4s in Antigenic Diversification

G4 motifs have been proposed to play roles not only in antigenic switching but also in the generation of antigenic diversity. So what are the molecular mechanisms by which these structures might induce recombination or mutation, and thus diversification of PQS-containing antigen-encoding genes? Recent research, using *S*. *cerevisiae* as a tractable tool with which to investigate G4 dynamics, has unearthed some possible mechanisms. Particularly informative is a reporter assay developed by the Nicolas laboratory that determines the frequency of size variants of the human G4-forming CEB1 minisatellite when inserted into a yeast chromosome. This work has established that it is replication fork stalling at unresolved G4s that leads to recombination and contraction or expansion of CEB1 repeats, and this is highly dependent on the Pif1 enzyme, an evolutionarily conserved helicase recently recognized to be a highly-efficient G4 helicase [19].

Interestingly, replication-dependent instability of G4 motifs was affected by the direction of the replication fork; it occurred only when the G4 was in the leading-strand template. The persistence of G4 structures on the leading-strand template led to Rad51/Rad52-dependent repair mechanisms, generating characteristic recombination intermediates and resulting in deletions at CEB1 repeats [60]. Determining the molecular detail of G4-dependent DNA transactions at such exquisite resolution may only be possible in a model organism like *S*. *cerevisiae*, but such work provides a model of how failure to resolve G4 structures can lead to error-prone recombinational repair that may result in the diversification of antigenic repertoires such as those encoded by *var*s or *vsg*s.

Elegant work in yeast has also provided clues about the possible role of G4s in Av occurring via epigenetic transcriptional switching. Paeschke et al. have showed that unresolved G4s can lead to changes in the epigenetic state of adjacent chromatin [46]. They introduced Pif1-binding G4 motifs into a nonessential arm of the yeast chromosome containing two selectable markers and assayed for gross chromosomal rearrangements or deletions (the term “deletion” normally refers to the loss of both markers, and is in contrast to a point mutation, which would affect only one marker). In wild-type strains, the rare loss of resistance to the selective agents did indeed result from loss of the whole chromosomal region. In Pif1-deficient strains, however, loss of resistance occurred much more frequently, and appeared to occur via epigenetic silencing: the sequences and positions of the selectable markers remained unchanged and their expression could be restored through deletion of *SIR2*, a histone-modifying deacetylase enzyme that is known to enforce epigenetic silencing. Thus, failure of G4 structures to be resolved resulted in epigenetic modifications near the G4 motifs. This may be because DNA synthesis becomes uncoupled from histone recycling mechanisms, leading to dysregulation of epigenetic status [44].

The high recombination rate of the HIV-1 provirus allows the virus to delay immune recognition and clearance, and G4 metabolism may be at play here too. Recombination hot spots and PQS have been found to correlate in the U3 promoter [51], *gag* [61] and cPPT [62] regions of the HIV-1 RNA genome, suggesting that G4s may contribute to this high mutation rate by enabling RT template switching during reverse transcription.

## Perspectives

**Fig 4:**
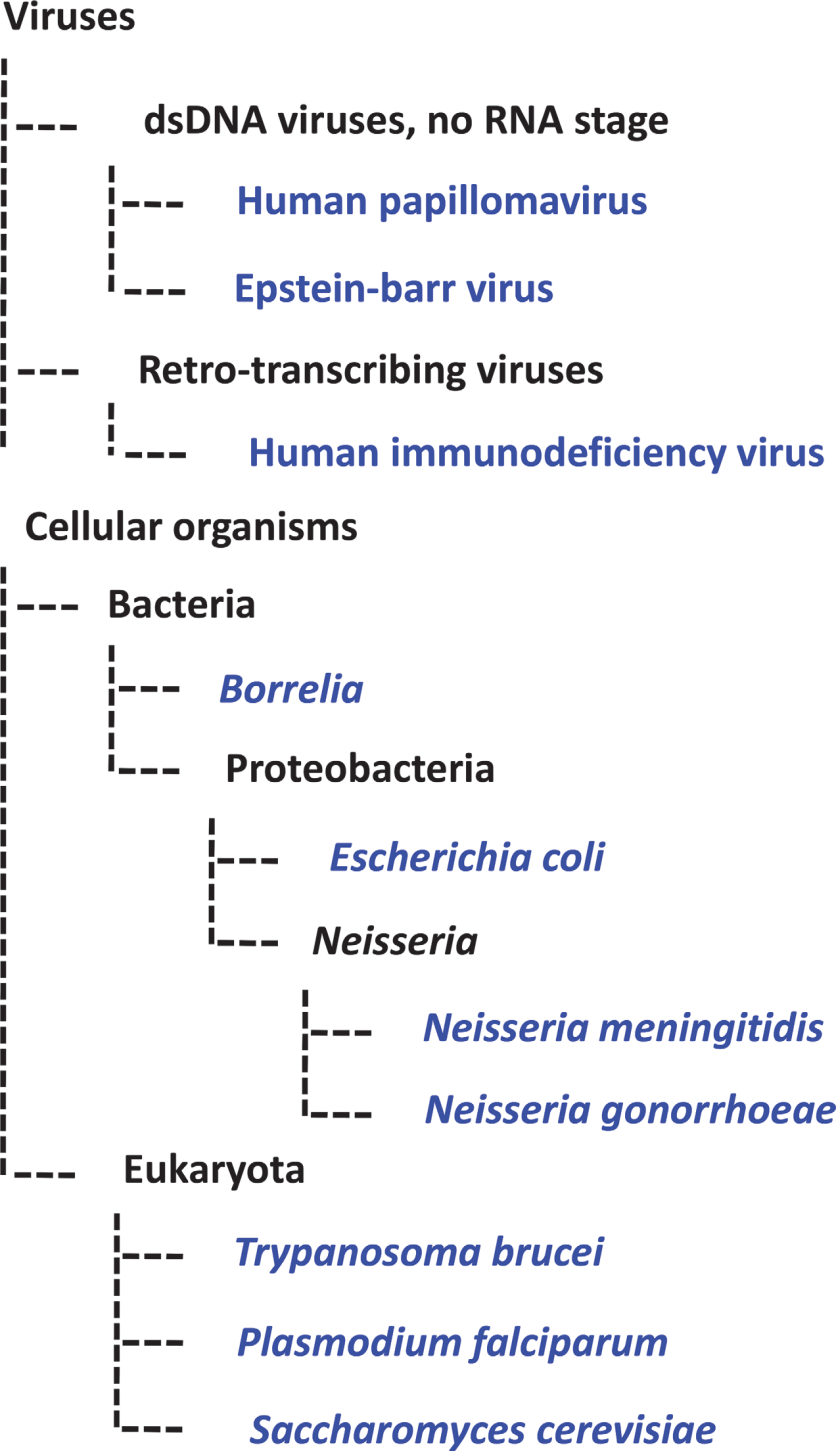
Phylogenetic tree schematic displaying the evolutionary relationships among the pathogens discussed in this review. The model systems *S*. *cerevisiae* and *E*. *coli* are also shown. Branches are not proportional to evolutionary distance.

G4 motifs offer disparate pathogens—from bacteria, to protozoa, to viruses (Fig. 4 and Table 1)—a means by which to regulate DNA and/or RNA dynamics underlying the common virulence phenotypes of antigenic variation and viral latency. Understanding the G4-mediated regulation of pathogen virulence may open the door to novel therapeutic interventions. Similar to anticancer strategies targeting G4s in telomeres and oncogenes, a pathogen’s RNA or DNA could be targeted by G4-specific ligands, and the high topological variety amongst G4 structures suggests that a high level of drug specificity could be achieved [63]. Indeed, the G4-stabilising ligand BRACO-19 is able to inhibit HIV-1 infectivity [49,56] and block the proliferation of EBV-positive cells in vitro [64].

**Table 1:** G-quadruplexes in pathogens.

	Pathogen	PQS/G4 location	G4 function	Virulence phenotype
Viruses	HPV	LCR; L2; E1; E4 [59]	(DNA transcription)	VL
	EBV	EBNA1 [57,58]	mRNA translation; Ag presentation	VL
	HIV-1	LTR; *nef* [49,54]	DNA/RNA transcription; TF binding; mutation rate	VL; AD
Prokaryotic pathogens	Lyme *Borrelia*	*vlsE* [32]	(Target for strand exchange)	AV
	*N. meningitidis*	*pilE* [31]	(DNA breakpoints; RF recruitment)	AV
	*N. gonorrhoeae*	*pilE* [24]	DNA breakpoints; RF recruitment	AV
Eukaryotic pathogens	*T. brucei*	*vsg*?	(Double-strand breaks)	AV
	*P. falciparum*	*Var* [39]	(DNA transcription; epigenetic silencing; recombinational repair)	AV; AD

Pathogens known or hypothesised to contain G-quadruplexes involved in virulence phenotypes. Brackets indicate hypothesised G4 functions which could influence the virulence phenotypes indicated. VL: viral latency, AV: antigenic variation, AD: antigenic diversity.

The wide range of pathogens using G4s in regulatory processes suggests that G4s may regulate similar processes in other pathogens. For example, in addition to *Neisseria* and *Borrelia* there are a number of other prokaryotic and eukaryotic pathogens that use controlled DNA-recombination-associated Av systems. Investigation of G4 dynamics in these pathogens, which include, among others, species of *Mycoplasma* [65], *Trypanosoma* [66] and *Babesia* [67], would merit an interesting line of investigation. Amongst viruses, the PQS found in the U3 promoter region of the HIV-1 proviral genome were found to be conserved in HIV-2 and simian immunodeficiency virus [51], indicating that G4s may affect the latency program of these viral species. Furthermore, in addition to EBV EBNA1 mRNA, PQS are present in the mRNA of a number of other gammaherpesviral genome maintenance proteins [57], which may also therefore be subject to G4-mediated translational control.

A highly G4-specific monoclonal antibody generated by phage display has recently been used to demonstrate the presence of G4s in the genomic DNA and cytoplasmic RNA of a range of human cells [68,69]. This antibody, or others similarly developed, could provide a useful tool with which to investigate pathogen G4 dynamics. Finally, it is worth noting that research on pathogen G4 dynamics is relevant to those pursuing G4s as anticancer targets. A number of cancers are associated with HPV and gammaherpesviruses such as EBV; therefore the development of new therapeutic targets based on G4s in these viruses also has the potential to reduce the burden of these malignancies.
